# Prevalence of depression and associated factors among patients with type 2 diabetes mellitus in eastern Sudan

**DOI:** 10.1186/s12888-021-03357-1

**Published:** 2021-07-06

**Authors:** Saeed M. Omar, Imad R. Musa, Maysoon B. Idrees, Ishag Adam

**Affiliations:** 1 Faculty of Medicine, Gadarif University, Gadarif, Sudan; 2 Royal Commission Hospital, Al Jubail Industrial City, Al Jubail Kingdom of Saudi Arabia; 3 Department of Obstetrics and Gynecology, Unaizah College of Medicine and Medical Sciences, Qassim University, Unaizah, Saudi Arabia

**Keywords:** Prevalence, Depression, T2DM, Risk factors

## Abstract

**Background:**

Diabetes mellitus (DM) represents a global health threat and burden. It is associated with medical and psychological complications, especially depression. Depression among patients with DM may affect the general prognosis. Hence, we conducted a cross-sectional study in Gadarif, eastern Sudan to evaluate the prevalence of depression and its associated factors among patients with type 2 DM (T2DM).

**Methods:**

We performed a cross-sectional study. Data on anthropometric parameters, demographic characteristics and blood glucose levels were collected via questionnaire. Depression was assessed using the Patient Health Questionnaire (PHQ-9).

**Results:**

Three hundred and fifty patients with T2DM were enrolled in the study and 205 (58.6%) were women. The median (interquartile range) age and duration of diabetes were 56.0 (14.0) years and 8 (8) years, respectively. The prevalence of depression in patients with T2DM was 35.6%. Logistic regression analysis showed significant associations between depression and rural residence (adjusted odds ratio [AOR] = 2.11, 95% confidence interval [CI] = 1.20–3.72), non-employee (AOR = 2.32, 95% CI = 1.34–4.00), co-morbidity (AOR = 2.35, 95% CI = 1.43–3.86) and obesity AOR = 2.19, 95% CI = 1.48–4.18).

**Conclusion:**

The prevalence of depression is high among Sudanese patients with T2DM. Rural residence, unemployment, co-morbidity and obesity are significant risk factors for developing depression among patients with T2DM.

## Introduction

The number of diabetes mellitus (DM) cases is rising globally, and its prevalence was estimated global population to be 9.3% (463 million people) of the in 2019 [1]. Its prevalence is expected to reach 10.2% (578 million people) by 2030 and 10.9% (700 million people) by 2045 [1]. Urban areas have a higher prevalence (10.8%) of DM than rural (7.2%) areas do, and high-income countries are affected more (10.4%) compared with low-income countries (4.0%) [1]. Type 2 DM (T2DM) patients represent the majority of patients suffering from DM, at 90% [1]. According to the International Diabetes Federation’s diabetes atlas for 2019, Sudan is reported among the countries that have a DM prevalence of more than 12% [1]. Recently, a community based research from Sudan demonstrated relatively higher prevalence rates of T2DM 20.8% of the studied population, from whom the newly diagnosed T2DM and uncontrolled T2DM, were 10 and 80% respectively [2].

Depression is growing as a major public health problem, exhibiting a global increase in its prevalence and incidence [3, 4]. This disorder poses a huge financial burden and affects many struggling national healthcare systems [5]. Several studies have reported a significant association between depression and DM [6, 7].

Mental health problems often co-occur in people with DM, as depression is twice as common in patients with DM compared with the general population, and it is associated with poor outcomes [8]. Patients with depressive symptoms have a 37–60% greater risk of developing DM compared with those without depressive symptoms [9–11]. Moreover, a reciprocal or bidirectional association between DM and depression has been documented in previous studies [8, 10, 11]. The association of depression and DM worsens the general outcome related to DM, impairs adherence to medical treatment [12], decreases quality of life [13] and increases fatality [14]. There are many identified risk factors that increase the possibility of depressive symptoms emerging among patients with DM; these include age, gender, urban residence, glycaemic control, smoking, obesity and family history of depression [15, 16].

Several previous studies have shown a high prevalence of depression among patients with DM in different populations in Africa [17–21]. However, few published data on depression and DM exist in Sudan [22, 23]. Patients with DM and depression are vulnerable groups and deserve more effort to shed light on and establish an integrated care system for feasibly and effectively managing such complex patients. Such interventions will be reflected positively in improving prognosis and quality of life. In fact, few published data address this issue in Sudan. Hence, the aims of the current study were to determine the prevalence of depression and its associated factors among patients with T2DM in eastern Sudan.

### Study area

Gadarif is one of 18 states in Sudan, and it is located in the eastern part of the country. It has the second-largest economy in Sudan. Gadarif State has a total population of 1,400,000 people, and it covers an area of approximately 75,263 km^2^. The state is located between latitudes 14 and 16 North and longitudes 35 and 36 East in the semi-desert tropics. The mosaic of the population mostly comprises Sudanese tribes engaged in agricultural and pastoral activities [24].

### Subjects and study design

A cross-sectional study was conducted in May–November 2019. Patients with T2DM were recruited from the outpatient diabetic clinic in Gadarif Diabetic Center. Gadarif diabetic Center is located in the Gadarif city which provides an outpatient services to all diabetic patients in the state who are already registered or referred to. The services were conducted by a team which include endocrinologist, surgeon, ophthalmologist, psychologist, medical laboratory technicians as well as services for foot care, health education and nutritional advices. All patients with type 2 DM who came to follow up during the study period to Gadarif diabetic centre were considered as the study population.

The systematic random sampling technique was used to select the participant who fulfilled the inclusion criteria. According to the Gadarif Diabetic Center records, there were 734 diabetic patients who visited the Center over six month’s period prior to the study. The sampling interval (≈ 2) was assumed by dividing the expected number (734) of diabetic patients to the calculated sample size (734/350 ≈ 2). Thus, eligible patients were interviewed for every 2 intervals until the required sample size (350) was reached.

Men and women aged 18 years and above were included after agreeing to participate in the research and giving written informed consent. Patients below the age of 18, those patients who had gestational diabetes or type 1 DM and those who refused to participate were excluded.

### Data collection and analysis

Well trained team including two general practitioners, a psychologist and two paramedical staff completed the questionnaire by direct face to face interview and conducted the physical examination under direct supervision of the primary investigators during a usual clinic visit. The minimum sample size was 350, calculated using the following formula:

$$ \frac{Z^2P\left(1-\right)}{d^2} $$

The prevalence of depression among DM patients in Sudan was 35.6% [23], with a 95% confidence interval (CI) and 5% precision. The collected data were analysed and correlated with the demographic data of the participants to determine whether the variables significantly correlated with depression among the recruited sample. The questionnaire was used to collect information on the following sociodemographic characteristics: age, sex, education (primary level, middle school and college level), employment (employed or unemployed), socioeconomic status (low, moderate or high), marital status (single, married or divorced), residence (urban or rural), alcohol consumption (never, current or former), smoking (never, current or former), comorbidities (hypertension, ischaemic heart disease, bronchial asthma, rheumatoid arthritis, malignancies and other), history of depression or depression among first-degree relatives. The questionnaire was also used to obtain a detailed history regarding DM, including the duration of DM, number of medications, insulin therapy, regular follow-up, presence of co-morbidities and complications related to DM. The participants’ weight and height were measured using standard procedures, and the body mass index (BMI) was computed using the following equation: weight (kg)/height (m^2^). The World Health Organization classification for BMI was followed which as either underweight (< 18.5 kg/m2), normal weight (18.5–24.9 kg/m2), overweight (25.0–29.9 (kg/m2), or obese (≥ 30.0 kg/m^2^) [25].

The study assessed depression using a validated previously used questionnaire which was the Sudanese version of Arabic translation of the Patient Health Questionnaire, (PHQ-9) [26]. The PHQ-9 is a nine-question questionnaire that screens for depression and assesses the severity. The PHQ-9 demonstrates high sensitivity (73%) and a very high specificity (98%) for diagnosing major depression, based on structured psychiatric interviews [27]. The PHQ-9 score ranges from 0 to 27, with each symptom scored on a scale of from 0 to 3 (0 = Not at all, 1 = several days per week, 2 = more than half of the days, 3 = nearly every day). The prevalence of depression was diagnosed with a cut-off score of ≥10, which is the most widely accepted to indicate positive cases of depression [28]. Patients who scored less than 10, 10–14, 15–19 and 20 or above were considered to have no depression, moderate, moderately severe, and severe depression, respectively [29]. The participants with a score ≥ 10 were classified as having depression in the further analysis.

### Blood glucose measurement

A sample of 3 ml of venous blood was collected from each participant by a university- graduated medical laboratory technicians, after adequately disinfected by alcohol swab and the procedure and technique were fully explained. The blood was then drawn in a vacuum blood collection tube containing EDTA, and the sample was transferred to the hospital laboratory department to measure HbA1c levels using an Ichroma machine (Republic of Korea). Glycaemic control was defined as recommended by the American Diabetes Association for no pregnant adults and the International Diabetes Federation [29] as Good glycaemic control was defined as an HbA1c level < 7.0%, where the uncontrolled is considered when HbA1c levels was ⩾7.0%.

### Statistical analysis

Data were analysed with a computer using SPSS for Windows (version 20.0). The chi-square test was used to compare proportions between participants with no depression and those who were diagnosed as having depression among patients with DM. Continuous parametric and nonparametric data were compared with the *t* test and Mann–Whitney *U* test, respectively, between the two groups (without depression and with depression). Logistic regression analyses were performed, using depression as the dependent variable. Independent variables (age, sex, BMI, marital status, education, job, socioeconomic status, rural or urban residency, smoking, alcohol consumption, history of depression, family history of depression, control of DM-associated co-morbidities) were entered into the model if their univariate *p* was < 0.20. Odds ratios (ORs) and 95% CIs were calculated, and a *p*-value < 0.05 was considered significant.

## Results

### General characteristics

Three hundred and fifty patients with T2DM were enrolled in the study. The median (interquartile range) age and duration of diabetes were 56.0 (14.0) years and 8 (8) years, respectively. Of the 350 participants, 205 (58.6%) were women, 261 (74.6%) were urban residents and 135 (38.6%) had an education level ≥ secondary level. The median (interquartile range) BMI was 25.9 (5.8) kg/m^2^, and 80% (22.9%) of participants were obese (Table 1). The prevalence of depression among patients with T2DM was 35.6%, ranging from mild (24.3%) to moderate (7.4%), moderately severe (2.2%) and severe depression (1.7%).

**Table 1:** Sociodemographic and clinical characteristics of patients with type 2 diabetes in eastern Sudan

Variable		Frequency	Proportion
Gender	Female	205	58.6
	Male	145	41.4
Residence	Urban	261	74.6
	Rural	89	25.4
Education level	< Secondary	215	61.4
	≥ Secondary	135	38.6
Marital status	Married	322	92.0
	Single/divorced	28	8.0
Job	Employed	141	40.3
	Non-employed	209	59.7
Income	Low	202	57.7
	Moderate	130	37.1
	High	18	5.1
Housing	With family	343	98.0
	With friends	7	2.0
Tobacco use	Never	302	86.3
	Current	19	5.4
	Former	29	8.3
Alcohol	Never	335	95.7
	Current	1	.3
	Former	14	4.0
Participant’s mental illness	Yes	24	6.9
	No	326	93.1
First degree mental illness	Yes	23	6.6
	No	327	93.4
Presence of co-morbidity	Yes	142	40.6
	No	208	59.4
Body mass index groups, kg/m^2^	Underweight	8	2.3
	Normal	134	38.3
	Overweight	128	36.6
	Obese	80	22.9
Uncontrolled diabetes	Yes	306	87.4
	No	44	12.6

There was no significant difference in the age, gender, residence, education, housing, marital status or alcohol and tobacco use between patients with T2DM who had depression and patients with T2DM who had no depression. In comparison with the patients who had no depression, patients who had depression were non-employed, had co-morbidity and were obese (Table 2). Logistic regression analysis showed that rural residence (adjusted OR [AOR] = 2.11, 95% CI = 1.20–3.72), non-employment (AOR = 2.32, 95% CI = 1.34–4.00), co-morbidity (AOR = 2.35, 95% CI = 1.43–3.86) and obesity (AOR = 2.19, 95% CI = 1.48–4.18) were associated with depression among patients with T2DM (Table 3).

**Table 2:** Comparing frequency (proportion) of the sociodemographic characteristics of patients who had depression and patients who had no depression in eastern Sudan

Variable		Patient with depression (***N*** = 87)	Patient who had no depression (***N*** = 263)	***p***
Gender	Female	51 (58.6)	154 (58.6)	1.000
	Male	36 (41.4)	109 (41.4)	
Residence	Urban	58 (66.7)	203 (77.2)	0.064
	Rural	29 (33.3)	60 (22.8)	
Education level	< Secondary	57 (65.5)	158 (60.1)	0.377
	≥ Secondary	30 (34.5)	105 (39.9)	
Marital status	Married	82 (5.7)	240 (8.7)	0.496
	Single/divorced	5 (94.3)	23 (91.3)	
Job	Employed	23 (26.4)	118 (44.9)	0.002
	Non-employed	64 (73.6)	145 (55.1)	
Income	Low	50 (57.5)	152 (57.8)	0.683
	Moderate	34 (39.1)	96 (36.5)	
	High	3 (3.4)	15 (5.7)	
Housing	With family	86 (98.9)	257 (97.7)	1.000
	With friends	1 (1.1)	6 (2.3)	
Tobacco use	Never	74 (85.1)	228 (86.7)	0.784
	Current	6 (6.9)	13 (4.9)	
	Former	7 (8.0)	22 (8.4)	
Alcohol	Never	83 (95.4)	252 (95.8)	0.804
	Current	0 (0.0)	1 (0.4)	
	Former	4 (4.6)	10 (3.8)	
Mental illness	Yes	4 (4.6)	20 (7.6)	0.464
	No	83 (95.4)	243 (92.4)	
First degree mental illness	Yes	5 (5.7)	18 (6.8)	1.000
	No	82 (94.3)	245 (93.2)	
Presence of co-morbidity	Yes	49 (56.3)	93 (35.4)	0.001
	No	38 (43.7)	170 (64.6)	
Body mass index (BMI) groups	Underweight	3 (3.4)	5 (1.9)	0.018
	Normal	28 (32.2)	106 (40.3)	
	Overweight	26 (29.9)	102 (38.8)	
	Obese	30 (34.5)	50 (19.0)	
Uncontrolled diabetes	Yes	78 (89.7)	228 (86.7)	0.577
	No	9 (10.3)	35 (13.3)

**Table 3:** Multivariate (non-adjusted and adjusted) analysis of the factors associated with depression among patients with type 2 diabetes in eastern Sudan

Variable		Non-adjusted			Adjusted		
		Odds ratio (OR)	95% confidence interval (CI)	*p*	OR	95% CI	*p*
Residence	Urban	Reference			Reference		
	Rural	2.23	1.25–3.98	0.006	2.11	1.20–3.72	0.009
Job	Employed	Reference			Reference		
	Non-employed	2.30	1.32–4.02	0.003	2.32	1.34–4.00	0.002
Presence of co-morbidity	Yes	2.56	1.51–4.34	< 0.001	2.35	1.43–3.86	0.001
	No	Reference			Reference		
Body mass index (BMI) groups	Underweight	2.46	0.52–11.63	0.255	2.75	0.59–12.71	0.193
	Normal	Reference			Reference		
	Overweight	0.99	0.52–1.86	0.982	0.98	0.52–1.83	0.959
	Obese	2.08	1.08–3.99	0.028	2.19	1.48–4.18	0.017

## Discussion

The study demonstrated that 35.6% of patients with T2DM had depression. This is exactly the same to the prevalence (35.6%) of depression reported in patients with T2DM in northern Sudan [23]. The prevalence in our study was considerably lower than the prevalence of depression (44%) in patients with T2DM in Omdurman, Sudan [22]. The prevalence of depression in this study was markedly higher than the global prevalence of depression (28%) among patients with T2DM [30]. At the same time, the prevalence of depression in this study was relatively lower than the prevalence rates of depression among patients with T2DM in Ethiopia (44.7%) [31] and in Rwanda (83.8%) [21]. However, the prevalence in our study was considerably higher than the prevalence rates of depression among patients with T2DM in Ghana (31.3%) [19] and Malawi (18%) [32]. The different prevalence rates obtained in these studies may be explained by the different methods used, including differences in the sample size, screening tools used for depression, different cut-off scores employed and sociodemographic statuses among the participants. A higher prevalence of depression among patients with DM may indicate a strong association between the two conditions, as both diseases might have a common aetiology [33]. Hence, the presence of DM increases the prevalence or risk for future depression, and at the same time, the presence of depression increases the potential risk for future DM [33]. In addition, chronic stress has behavioural consequences as a result of stress hormone secretion, which increases the risk for both depression and DM [34]. Interestingly, patients with either DM or depression are vulnerable to neurodegenerative processes that are associated with structural changes in the prefrontal cortex and hippocampus [35, 36]. The results of these changes are cerebral atrophy and recurrent infarcts and cerebral blood flow changes of both hypo- and hyper-perfusion [35, 36].

This study showed that rural residents with T2DM had a 2.11 times higher risk of depression. This was in accordance with the results obtained in Kenya, where depression was prevalent among rural people with DM [37]. This may reflect rural–urban health disparities in Africa because rural dwellers were more likely to be older, have lower education levels, have poverty and have less cognitive functioning compared with urban dwellers [38]. In contrast, depressive symptoms were more prevalent among urban residents with T2DM in Guinea [39]. A higher risk of depression may be related to the interaction between urbanicity, the socio-ecological environment and mental health.

Our study showed that the unemployed were at higher risk for depression among patients with T2DM. Several studies conducted in African countries, such as South Africa [40], Guinea [39] and Rwanda [21], have shown that unemployment is a significant risk factor for depression in patients with T2DM.

In the current study, obese patients had a 2.19 times higher likelihood of developing depression among patients with T2DM. Similar findings of significant associations between obesity and depression in participants with T2DM were observed in Ethiopia [31] and Guinea [39]. In contrast, another study failed to show an association between obesity and depression among patients with T2DM in Ghana [19]. The association between obesity and depression may be explained in that the two diseases share common biological pathways [41]. The depression–obesity link involves genetics, alterations in systems involved in homeostatic adjustments and brain circuitries integrating homeostatic and mood regulatory responses [41]. Moreover, the homeostatic adjustments depend on the hypothalamus–pituitary–adrenal axis, immuno-inflammatory activation and neuroendocrine regulators of energy metabolism, such as insulin, leptin and the gut microbiome [41].

There was no association between depression and age in the current study. Similar findings in previous studies showed no association between depression and age in central Sudan, Omdurman [22], Tanzania [20], Ethiopia [42] and Nigeria [43]. A meta-analysis of patients with T2DM revealed that depression was more prevalent in subjects younger than 65 compared with older people [30]. Depression among older patients with DM may be a consequence of the neurodegenerative processes that potentiate the risk of depression [35, 36].

Our study showed no significant association between gender and depression among patients with T2DM. A similar lack of association was observed among patients with T2DM in northern [23] and central Sudan [22], as well as in Malawi [32]. In contrast, gender was reported as a significant risk for depression among patients with T2DM in South Africa [40], Guinea [39], Ghana [19] and Rwanda [21]. A predominance of female gender is proposed by *cognitive vulnerability–stress models* of depression, and this is characterised by higher levels of negative cognitive style and stress in girls than boys beginning in early adolescence [44].

Our study showed that glycaemic control was not a significant risk for developing depression among patients with T2DM. This is a similar outcome to the non-significant association of depression and glycaemic control using fasting blood sugar as reference in patients with T2DM found in Ethiopia [31, 45] and subjects with DM in Nigeria [43]. In contrast, a significant difference between depression and poor glycaemic control (HbA1c > 7%) among patients with DM was documented in Sudan [23], Guinea [39], Ghana [19] and South Africa [40]. It is recognised that a high level of HbA1c is a significant predictor of hippocampal volume [46], and this leads to similar neurodegenerative processes to those associated with depression [36].

### Limitations of the study

The limitations of this study are that it is a facility based which may not reflect the prevalence among the community diabetic patients and the cross-sectional nature of the study. In addition, the use of the PHQ-9 psychiatric scale is not the gold standard scale to assess depression.

## Conclusion

The prevalence of depression is high among Sudanese patients with T2DM. Rural residence, unemployment, co-morbidity and obesity appear to be significant risk factors for developing depression among patients with T2DM. Hence, efforts to minimise the burden of depression and improve the future of DM in Sudan are highly recommended.

## Data Availability

The datasets used and/or analysed during the current study are available from the corresponding author on reasonable request.

## Abbreviations

BMI: Body mass index
OR: Odds ratio
CI: Confidence interval
PHQ: Patient health questionnaire
T2DM: Type 2 diabetes mellitus
